# An Iterative Closest Points Algorithm for Registration of 3D Laser Scanner Point Clouds with Geometric Features

**DOI:** 10.3390/s17081862

**Published:** 2017-08-11

**Authors:** Ying He, Bin Liang, Jun Yang, Shunzhi Li, Jin He

**Affiliations:** 1 Shenzhen Graduate School, Harbin Institute of Technology, Shenzhen 518055, China; 2 Department of Automation, Tsinghua University, Beijing 100084, China; he-j15@mails.tsinghua.edu.cn; 3 Shenzhen Graduate School, Tsinghua University, Shenzhen 518055, China; yangjun603@mail.tsinghua.edu.cn (J.Y.); lisz15@mails.tsinghua.edu.cn (S.L.)

**Keywords:** ICP registration, geometric features, point clouds

## Abstract

The Iterative Closest Points (ICP) algorithm is the mainstream algorithm used in the process of accurate registration of 3D point cloud data. The algorithm requires a proper initial value and the approximate registration of two point clouds to prevent the algorithm from falling into local extremes, but in the actual point cloud matching process, it is difficult to ensure compliance with this requirement. In this paper, we proposed the ICP algorithm based on point cloud features (GF-ICP). This method uses the geometrical features of the point cloud to be registered, such as curvature, surface normal and point cloud density, to search for the correspondence relationships between two point clouds and introduces the geometric features into the error function to realize the accurate registration of two point clouds. The experimental results showed that the algorithm can improve the convergence speed and the interval of convergence without setting a proper initial value.

## 1. Introduction

The 3D point cloud of the object surface can be obtained by optical equipment such as laser scanners, which can provide the basis for the establishment of the 3D model of the object. However, it is impossible to obtain all the point cloud information of the object at the same viewpoint because the 3D scanning device has a limitation on the field of view or because of the complex geometry of the object itself. In order to obtain the complete point cloud data of the measured object, it is necessary to integrate the part of the surface point cloud data obtained from different angles [1]. The purpose of point cloud registration is to find a 3D rigid body transformation, so that the 3D coordinates of the point cloud at different angles can be correctly matched and overlapped. In reverse engineering, computer vision and graphics databases based on graphical searching, point cloud registration has a wide range of applications.

How to register the scattered point cloud of these large-scale data quickly and accurately is a research hotspot of researchers at present. The most prominent contribution is the Iterative Closest Points (ICP) algorithm proposed by Besl [2]. In this method, the transformation parameters of two point sets are calculated through the relationship between the corresponding matching points of two point sets to satisfy the given convergence precision, and finally the translation and rotation parameters between the two points are obtained to complete the registration process. However, there are some problems with the traditional ICP algorithm [3], where the initial value of the iteration should be determined when the first step of the ICP algorithm is performed. The selected initial value will have major effect on the final registration result. If the selection of the initial value is not appropriate, the algorithm may lead to a local optimum, so that the iteration cannot converge to the correct registration result.

To address the problems of the ICP algorithm, many improved algorithms based on ICP framework have been proposed by researchers because of its outstanding advantages. To summarize, each improved algorithm improves the performance by adjusting one or more of the four steps of the original algorithm. *Point selection*. The ICP algorithm needs to find the nearest point of each point in the current point set at the point of the other point set in each iteration, so the computation is complicated. This process can be accelerated by down-sampling the original point set [4,5]. *Finding corresponding points*. The ICP algorithm needs to find the nearest point from another point set as the corresponding point of the current point. By using the kd-tree data structure, projection, invariant feature search algorithm [6,7,8,9,10,11,12,13,14,15] to effectively find the corresponding relationship between the two point sets, we can speed up the search process and improve the corresponding precision. *Point pair exclusion*. The appropriate error points in the exclusion method can improve the point cloud data stitching accuracy and stability [16]. *Specifying error metrics function and minimizing errors*. Specifying the appropriate error metric function can improve the accuracy of point cloud registration [17,18]. 

This paper focuses on the ICP algorithm for registration of 3D point cloud with geometric features. Cheng et al. [19] combined feature lines and corner points to register point cloud data semiautomatically. Nevertheless, this method mainly focuses on the extraction of geometric features from the cuboid-shaped buildings. A similar solution was proposed by Wu [20], who considered building roof features. Both methods have a low degree of automation. Hansen [21] proposed an automatic registration method by identifying the correspondences between extracted feature lines. Then orientation histograms were applied for the rotation, and generate-and-test scheme was used for the translation parameters. This method does not need prior knowledge. However, many useless feature lines from the point cloud were also extracted. 

In order to minimise the search space for correspondence between two point clouds and to increase the accuracy in the selection of the corresponding points, Rabbani [22], Nrenner [23] and Barnea [24] have used geometric features. Sharp [9] proposed to use either spherical harmonics or the second order momentum to minimize the error to find the correspondence of 3D range camera datasets. Hao [25] proposed a variant of the extended Gaussian image based registration algorithm for point clouds with surface color information. Sharp [9] used invariant features in the ICPIF algorithm to obtain correspondences. Aiger [10] proposed the 4-Point Congruent Sets (4PCS) algorithm based on the affine invariant ratio of four congruent points on the plane. Experimental results show that the 4PCS algorithm can effectively improve the robustness of point cloud data surface stitching. Ge [11] adopted an intrinsic geometric approach in which geodesic distance is exploited as the key factor to establish stable correspondences between two scans on the basis of the 4PCS algorithm. Bae [12] proposed Geometric Primitive ICP (GP-ICP) algorithm based on curvature and normal rate of change. Experiments showed that GP-ICP could increase the convergence region. 

Although these feature-based ICP methods [9,26] increase the accuracy in selecting corresponding points and the efficiency of the algorithm, a registration method with large convergence region is still to be developed [27]. In this paper, an algorithm based on point cloud features is proposed. The method uses the geometrical features of the point cloud to be registered, such as curvature, surface normal and point cloud density, to search the correspondence relationship between two point clouds and introduce the geometric features into the error function, to achieve accurate registration of the two point clouds. The method does not need to set a proper initial value, which can avoid the ICP algorithm into a local extremum and has a high convergence rate.

The rest of this article is organized as follows: the second part introduces ICP algorithm. The third part introduces the calculation of the geometric features of the point cloud curvature, normal and density. The fourth part details the ICP algorithm based on point cloud features. In the fifth part, the algorithm is verified by the simulation.

## 2. ICP Algorithm

Iterative closest point (ICP) registration is an accurate and reliable method for registration of free form surfaces [2]. ICP algorithm is used to find the rigid transformation T between the target point set S and the reference point set M so that the two matching data satisfy the optimal match under some kind of metric criterion. Assuming that the coordinates of the target point set S are $\left\{S_{i}|S_{i}\in R^{3},i=1,2,\cdots ,N_{s}\right\}$, the coordinates of the reference point set M are $\left\{M_{i}|M_{i}\in R^{3},i=1,2,\cdots N_{M}\right\}$, in the k-th iteration, the coordinates of the corresponding point corresponding to the coordinates of the point set S are $\left\{M_{i}^{k}|M_{i}^{k}\in R^{3},i=1,2,\cdots ,N_{M}\right\}$. The transformation matrix between S and $M^{k}$ is calculated and the original transform is updated until the distance between the data is less than the given threshold τ. The ICP algorithm steps are as follows:

(1) Calculate the corresponding point $M_{i}^{k}\in M^{k}$ in the reference set M so that $\left\|M_{i}^{k}-S_{i}^{k}\right\|=\min$;
(2) Calculate the rotation matrix $R^{k}$ and the translation vector $T^{k}$ so that $\sum_{i=1}^{N}{\left\|R^{k}S_{i}^{k}+T^{k}-M_{i}^{k}\right\|}^{2}=\min$;
(3) Calculate $S^{k+1}=\left\{S_{i}^{k+1}|S_{i}^{k+1}=R^{k}S_{i}^{k}+T^{k},S_{i}^{k}\in S\right\}$;
(4) Calculate $d^{k+1}=\sum_{i=1}^{N}{\left\|S_{i}^{k+1}-M_{i}^{k}\right\|}^{2}$;
(5) If $d^{k+1}$ is not less than the given τ value, return (1) until $d^{k+1}<\tau$ or iterations k is greater than the preset maximum number of iterations.

The average complexity of ICP algorithm is O(nlogn) (where *n* is the number of point cloud points), and it can be effectively converged to a local minimum. The estimation of a proper initial transformation is necessary, and ICP algorithm assumes that all points of the target point set correspond to the set of reference points.

## 3. Geometric Features of Point Clouds

Geometric features such as curvature, surface normal, and density can reflect the most basic geometric shapes of point clouds, which are critical to express the characteristics of point clouds. In this paper, we use the geometrical parameters related to the coordinates of point cloud to calculate the features of each data point. Assuming that the point cloud data set is $G=\left\{g_{i}\right\}$, i=1,⋯N, where $\left(x_{i},y_{i},z_{i}\right)$ are the 3D coordinates of the point cloud $g_{i}$, N is the number of point cloud data points.

### 3.1. Curvature 

Curvature is an important basis for feature recognition. The value of the curvature reflects the concavo-convex degree of the point cloud surface. The sharp features of the point cloud have a relatively large curvature. On the other hand, the non-feature parts of the point cloud have a relatively small curvature. In this paper, we use the method of [28] to estimate the normal and curvature of the data points by analyzing the covariance of the k neighboring points. For the point cloud data set G, the neighboring points covariance of the given point $g_{i}$ is analyzed and the covariance matrix is solved. The eigenvector direction corresponding to the minimum eigenvalue is defined as the normal of the point. Then, according to the surface change of the point in local region, the curvature can be estimated:

(1) $$C_{i}={\left[\begin{array}{c} g_{i1}-{\bar{g}}_{i}\\ \cdots \\ g_{ik}-{\bar{g}}_{i} \end{array}\right]}^{T}\left[\begin{array}{c} g_{i1}-{\bar{g}}_{i}\\ \cdots \\ g_{ik}-{\bar{g}}_{i} \end{array}\right]$$

where $C_{i}$ is a semi-positive definite three-order symmetric matrix, ${\bar{g}}_{i}$ is the center of the neighboring points of point $g_{i}$. Then the three eigenvalues of the matrix $\lambda_{1},\lambda_{2},\lambda_{3}$ and its corresponding unit eigenvectors $e_{1},e_{2},e_{3}$ can be calculated. Without loss of generality, we assume that $\lambda_{1}\leq \lambda_{2}\leq \lambda_{3}$. $\lambda_{1}$ describes the change value of the surface along the normal direction, then the normal direction $n_{i}=e_{1}$ of vertex $g_{i}$. The surface variation of $g_{i}$ can be expressed as follows:

(2) $$\tau_{i}=\frac{\lambda_{1}}{\left(\lambda_{1}+\lambda_{2}+\lambda_{3}\right)}$$

The curvature $H_{i}$ [29] of the point cloud model in the data point $g_{i}$ can be approximated as a surface variation $\tau_{i}$.

### 3.2. Angle between the Data Point Normal Direction and the Neighboring Points Normal Direction

The change of normal angle is also an important index to measure whether the surface is curved or straight. We assume that data point $g_{i}$ is a random point of the point cloud model G, and $g_{j}$ is a neighboring points of $g_{i}$. The normal directions of $g_{j}$ and $g_{i}$ are respectively $n_{g_{j}}$ and $n_{g_{i}}$. The normal angle cosine between $g_{i}$ and $g_{j}$ can be express as the following equation:

(3) $$\cos\theta_{g_{i}g_{j}}=\frac{n_{g_{i}}\cdot n_{g_{j}}}{\left|n_{g_{i}}\right|\left|n_{g_{j}}\right|}$$

where the value range of $\theta_{g_{i}g_{j}}$ is [0,π].

The angle parameter between data point and neighboring points is calculated by summing all the normal angle among its neighboring points:

(4) $$\omega_{a}\left(g_{i}\right)=\sum_{g_{j\in M\left(g_{i}\right)}}\theta_{g_{i}g_{j}}$$

**Figure 1:**
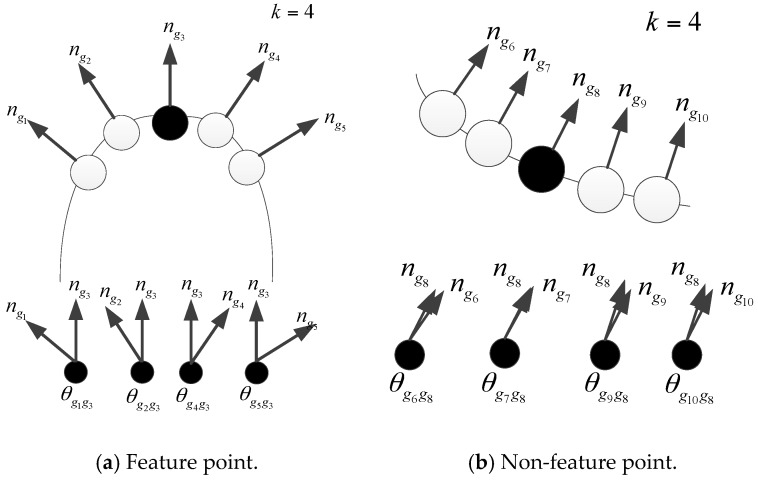
Feature point and the normal angle of its neighboring points.

Normal direction angles between each data points (including feature points and non-feature points) and its neighboring points are given in Figure 1, where $g_{3}$ is the feature point and the number of neighboring points are k=4. Moreover, the curve degree at feature point $g_{3}$ is relatively large, and the normal direction angles between feature point $g_{3}$ and its neighboring points are also relatively large.

By using the angle parameters, properly considering the impact of all the neighboring points on the bending degree of the data points $g_{i}$. If $\omega_{a}\left(g_{i}\right)$ is larger, the surface bending degree of data point $g_{i}$ and its neighboring points will be relatively large, and the neighbor region of data point $g_{i}$ will be more likely feature region. On the other hand, if $\omega_{a}\left(g_{i}\right)$ is smaller, the surface of the model will be relatively smooth, and the neighbor region of data point $g_{i}$ will be more likely non-feature region.

### 3.3. Feature Parameters

In this paper, we use the method [30,31] to integrate the surface curvature $H_{i}$ and the normal angle parameter $\omega_{a}\left(g_{i}\right)$ of the data point $g_{i}$ obtained as the dimensionless parameter values, and define the characteristic parameters of the data point $g_{i}$ as follows :

(5) $$\omega \left(g_{i}\right)=\lambda_{H}H_{i}+\omega_{a}\left(g_{i}\right),$$

where $\lambda_{H}$ is the surface curvature coefficient.

According to the above equation, the larger the surface curvature, the larger the normal angle parameter, the more likely the data points are feature points, so the surface curvature and the normal angle parameters are proportional to feature parameters.

After analyzing different data, the surface curvature coefficient $\lambda_{H}$ has a great influence on the calculation result. The number of neighboring points depends on the density of the point cloud data and the uniformity of the distribution. When the point cloud density is large, the value may be smaller. Generally, the value is 10–30. In this paper, the surface variation coefficient $\lambda_{H}$ = 200, the number of neighboring points k = 10. 

### 3.4. Geometric Features Detection Rsults

**Figure 2:**
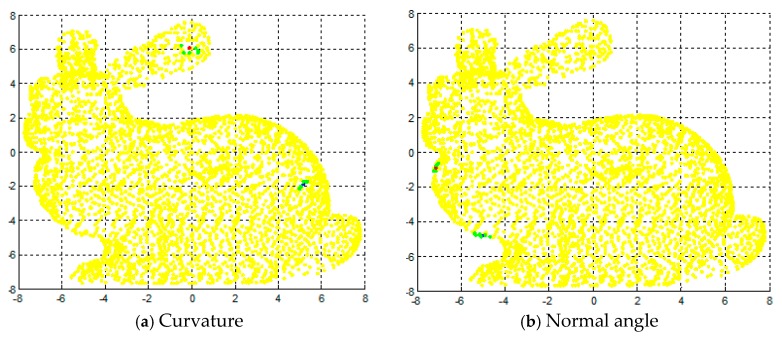
The detection results of feature points and non-feature points.

**Figure 3:**
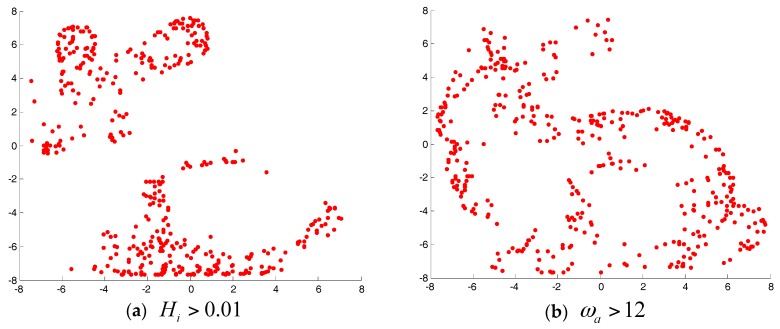
The detection results of three geometric features.

**Figure 4:**
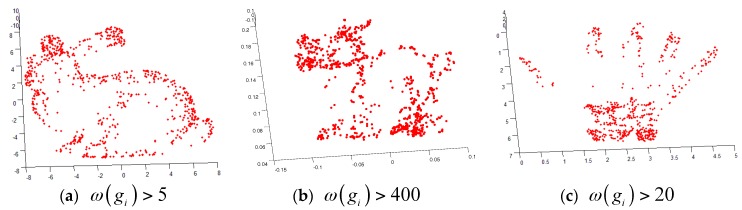
The results of calculation based on point cloud features.

The purpose of this experiment is to verify the effectiveness of the feature detection algorithm described in this paper. Firstly, we analyzed the effect of two kinds of geometric feature using standard point cloud data from the Stanford University Graphics Lab "bunny". In this paper, we used MATLAB to perform uniform sampling of point cloud data before experiment. After sampling, the data points were reduced to 3951. We selected six neighborhood points. Figure 2 shows the feature points and non-feature points of the geometric features of the three point clouds. In the map, red spots are feature points, blue spots are non-feature points, and green spots are neighborhood points. In Figure 2a, the surface curvature of feature point is 0.1441, the surface curvature of non-feature point is 0.00015; in Figure 2b, the normal angle of feature point is 15.5622, the normal angle of non-feature point is 0.1235. As shown in Figure 2 and Figure 3, this paper describes two kinds of geometric feature parameters that can reflect the features of point cloud area. Figure 4 shows the detection results of the geometric features of the two kinds of point clouds.

Next, we evaluated the performance of the above algorithm using standard point cloud data from the Stanford University Graphics Lab such as “bunny”, “dragon” and “hand”. In this paper, we used MATLAB to perform uniform sampling of point cloud data before experiment. After sampling, the data points of “bunny”, “dragon” and “hand” were reduced to 3951, 4377 and 3274, respectively. We selected the surface variation coefficient $\lambda_{H}$ = 200, the number of neighboring points k = 10. Figure 4 shows the results of “bunny”, “dragon”, and “hand” feature detection. It can be seen that the head, legs, tail and fingertip of the model are feature regions. In these parts, the corresponding feature parameters are relatively large. However, the body part of the model is non- feature region, and the corresponding feature parameters are relatively small, which are not shown in figure.

## 4. ICP Using Geometric Features

The ICP algorithm is an iterative algorithm, which requires a proper initial value and two point cloud approximate alignments to prevent the algorithm from falling into a local extremum, to ensure the accuracy, convergence speed and stability of the algorithm, but in the actual point cloud matching process, it is difficult to ensure compliance with this requirement. In these cases, geometrical features such as curvature, surface normal and point cloud density can provide additional information to restore the corresponding relationship between two point clouds. In this paper, we proposed a method to search the corresponding relationship between two point clouds by geometric features, and avoid the local extremum of the ICP algorithm so as to realize the registration of two point clouds.

**Figure 5:**
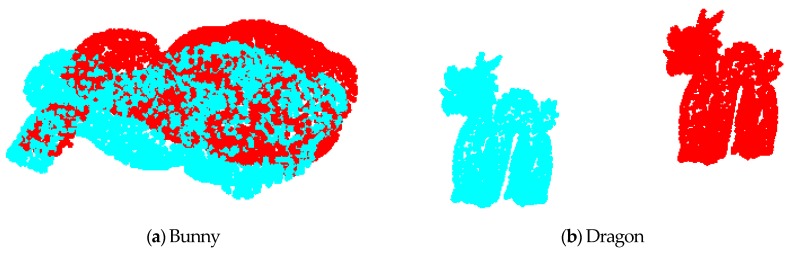
The original point cloud data before matching.

The geometric features can be calculated directly from the scattered point cloud by the method mentioned in this paper, which uses geometric features to achieve the initial match of the two point clouds. For this method, there is no need to set a proper initial value. In the matching algorithm, the amount of point cloud data is very large, which limits the speed of the registration algorithm. Geometric feature points with higher feature parameters may have more valuable information because they may be edges or corners. Therefore, in order to speed up the algorithm, in the initial match, we only considered the feature parameters with higher geometric feature points. Our algorithm is described as follows:

(1) Find the nearest k data points for each data point as neighboring points in the two point clouds. The geometric features and feature parameters of each point will be calculated.
(2) Select the initial sample points $\left\{p_{i}\right\},p_{i}\in S,M$ of two point clouds. The selected initial sample points satisfy $\omega \left(p_{i}\right)>\tau_{gf}$, where $\omega \left(p_{i}\right)$ is the feature parameter of point cloud data point $p_{i}$, and $\tau_{gf}$ is the feature parameter threshold. The selection of $\tau_{gf}$ value should be based on different point cloud data (For example , we selected $\tau_{gf}=5$ in Figure 5a).
(3) Find the corresponding points of two point clouds. If $\left|H\left(p_{i}^{s}\right)-H\left(p_{j}^{M}\right)\right|\leq \tau_{curvature}$ and $\left|\omega_{a}\left(p_{i}^{s}\right)-\omega_{a}\left(p_{j}^{M}\right)\right|\leq \tau_{normal}$ are satisfied, point $p_{i}^{s},p_{i}^{s}\in S$ is the corresponding point of point $p_{j}^{M},p_{j}^{M}\in M$, where H, $\omega_{a}$ are the surface curvature and normal angle respectively of the neighbor point, and $\tau_{curvature}$ and $\tau_{normal}$ are the surface curvature threshold and the normal angle threshold respectively.
(4) Calculate the rigid transformation $R^{1},T^{1}$ and obtain the transformed $S^{1}$, where $S^{1}=R^{1}S+T^{1}$, where the superscript 1 represents the initial value of the iteration.
(5) Find two corresponding points and calculate the matching error. Find each data point $S_{i}^{k}$ of $S^{k}$ from point cloud M of its nearest data point $M_{j}^{k}$ as its corresponding point. Calculate the matching error $d^{k}=\sum_{i=1}^{N}{\left\|S_{i}^{k}-M_{j}^{k}\right\|}^{2}+H\left(S_{i}^{k},M_{j}^{k}\right)+\omega_{a}\left(S_{i}^{k},M_{j}^{k}\right)$ based on the resulting corresponding point, where k is the number of iterations.
(6) According to the new point cloud correspondence relationship, obtain rigid transformation $R^{k},T^{k}$, and obtain $S^{k}$ after the transformation.
(7) Repeat step 5 until the two match errors are less than the threshold $\tau_{d}$ or the maximum number of iterations.

By comparing the geometric features of the point clouds, we realized the initial match of two point clouds. The initial match is close to the correct value, thus reducing the number of subsequent iterations, which not only speeds up the algorithm running speed but also avoids the algorithm into a local extreme.

## 5. Experimental Results

In order to verify the validity of the ICP algorithm based on point cloud features, we performed two sets of experiments in this paper. Experiment 1 analyzed the accuracy and speed of the algorithm, while Experiment 2 analyzed the algorithm's immunity to noise.

### 5.1. Accuracy and Speed Assessment

In order to evaluate the accuracy of the ICP algorithm based on point cloud features, this section compares the algorithm with the performance of the main variants of ICP algorithms (ICP algorithm based on quaternion and ICP algorithm based on a kd-tree). We used standard scanning point cloud data “bunny” and “dragon” provided by the Stanford University Graphic Laboratory to evaluate the performance of the algorithm. In this paper, the point cloud data is down-sampled by MATLAB (After sampling, the bunny is 3951 and dragon is 4377), and the point cloud position is generated randomly, the rotation matrix and translation vector are respectively:

$$\begin{array}{l} R_{b}=-\left[\begin{array}{ccc} 0.9363 & -0.3129 & 0.1598\\ 0.3205 & 0.9470 & -0.0237\\ -0.1439 & 0.0734 & 0.9869 \end{array}\right],\;T_{b}=\left[\begin{array}{c} 2\\ 0.6\\ 0.7 \end{array}\right]\\ R_{d}=\left[\begin{array}{ccc} 0.9974 & -0.0599 & 0.04\\ 0.06 & 0.9982 & 0\\ -0.0399 & 0.0024 & 0.9992 \end{array}\right],\;T_{d}=\left[\begin{array}{c} 0.4\\ 0\\ 0.2 \end{array}\right] \end{array}$$

**Figure 6:**
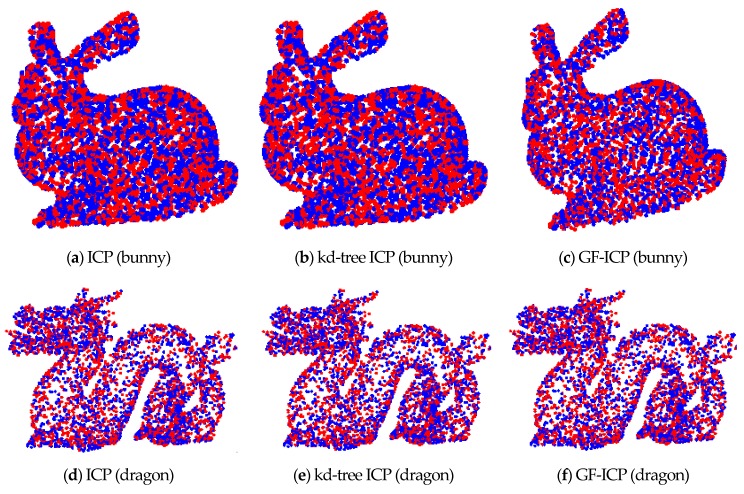
The results of three point cloud matching algorithms.

Figure 5 is the original point cloud data before matching. Figure 6 shows the results of point cloud matching by the three algorithms. Among them, the value of $\tau_{curvature}$ and $\tau_{normal}$ in the GF-ICP algorithm is set to 0.0001 and 0.1, the value of $\tau_{d}$ is 0.00001. By registration of the point cloud, the registration results of the three algorithms of bunny are $R_{bp}=R_{b},T_{bp}={\left[\begin{array}{ccc} 2.0323 & 0.6173 & 0.6990 \end{array}\right]}^{T}$; and the registration results of the three algorithms of dragon are $R_{dp}=R_{d},T_{dp}={\left[\begin{array}{ccc} 0.407 & 0.024 & 0.1839 \end{array}\right]}^{T}$. The two ICP algorithm matching error of bunny is 7 × 10^−6^ m, while the matching error of dragon is 9.9 × 10^−8^ m. The GF-ICP algorithm matching error of bunny is 1.6 × 10^−6^ m, while the matching error of dragon is 1.7 × 10^−8^ m. The results show that the ICP algorithm based on point cloud features in this paper achieves a better point cloud matching effect than the other two algorithms.

**Figure 7:**
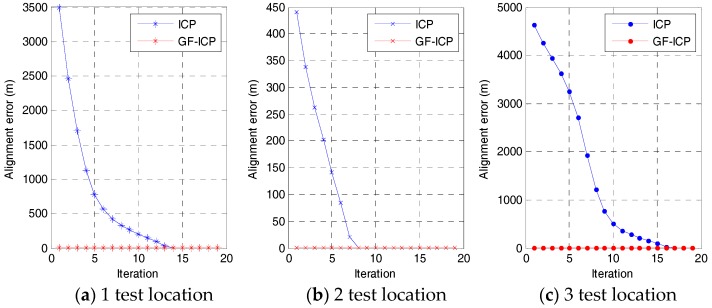
Comparison of convergence rate and matching error.

**Figure 8:**
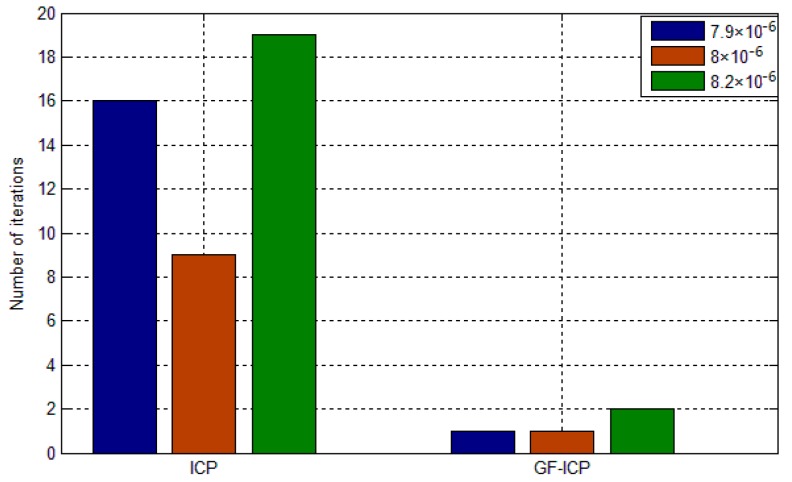
The number of iterations required by the two methods under the same accuracy.

In order to evaluate the speed of the ICP algorithm based on point cloud features, we randomly generated three sets of positions for the “bunny” point cloud data by using MATLAB. Figure 7 and Figure 8 shows the comparison of the convergence rate and matching error between the ICP algorithm based on quaternion and the ICP algorithm based on point cloud features under the same test conditions. From the figures, it is obvious that, when the algorithms achieve the same matching error, the convergence rate of the algorithm we proposed in this paper is better than that of the ICP algorithm in the three tests. Moreover, only two iterations are needed to achieve a stable low error state.

In the above experimental state, the run times of the ICP algorithm based on the quaternion, the ICP algorithm base on kd-tree and the algorithm based on point cloud features are listed in Table 1. It can be seen from the table that the algorithm proposed in this paper is slower than the ICP algorithm based on k-d-tree and has a slight advantage over the ICP algorithms based on the quaternion. Because the algorithm proposed in this paper has obvious advantage over the iterations, but because it requires computation the geometric feature of the point cloud, the running time is slightly slower than the ICP algorithm base on k-d-tree, but the running time of this algorithm shows that the algorithm is useful in practice.

In order to evaluate the convergence of the ICP algorithm based on point cloud features, we used MATLAB to generated 9 sets of positions for “bunny”, and each group data translation is $\left[\begin{array}{ccc} 1.6 & 0 & 0.4 \end{array}\right]$, rotating is α= 0 rad, β= 0.16 rad, γ= 0.24 rad. Table 2 lists the point cloud registration error and the number of iterations required by the ICP algorithm based on the quaternion and the algorithm proposed in this paper. 

It can be seen from the table that for the ICP algorithm based on the quaternion, the increase of the initial position of the point cloud will lead to a gradual increase in the required number of iterations. When the initial position reaches a certain value, the ICP algorithm will get into a local extremum, which cannot achieve two point cloud registration. However, with the algorithm we propose, the number of iterations required is less under the same matching error, and even when the initial position of the two point cloud to be registered is larger, it can also achieve a stable low matching error.

### 5.2. Analysis of Partially Overlapped Registration Results

**Figure 9:**
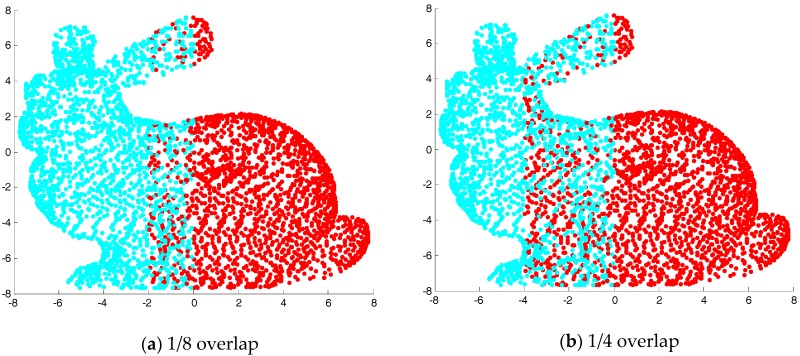
The overlap of point clouds.

**Figure 10:**
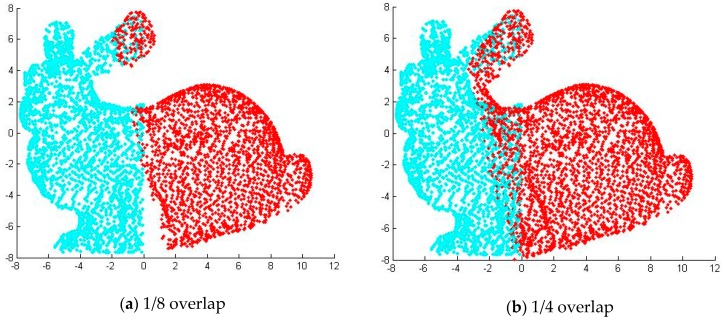
The original point cloud data before matching.

**Figure 11:**
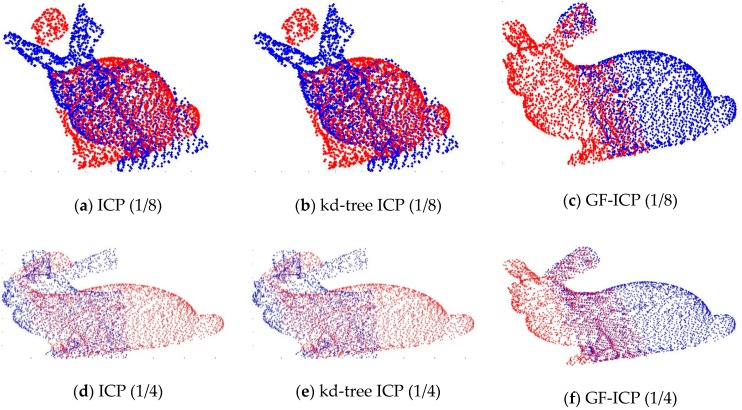
Partial cloud overlap registration results.

In order to verify the registration effect of the ICP algorithm based on point cloud features, the “bunny” point cloud data are separated from the middle by two sets of overlapping effects, as shown in Figure 9. We used MATLAB to randomly generate a rotation and translation for the second half of the “bunny” point cloud, as shown in Figure 10. Figure 11 shows the registration results of two ICP algorithms and the algorithm proposed in this paper under the same test conditions. It can be seen from the results that the ICP algorithm based on quaternion and the ICP algorithm based on kd-tree are consistent in the registration results, and none of them have been registered. However the algorithm proposed in this paper achieves better registration results in the presence of two different cloud overlap.

### 5.3. Analysis of Immunity to Noise

**Figure 12:**
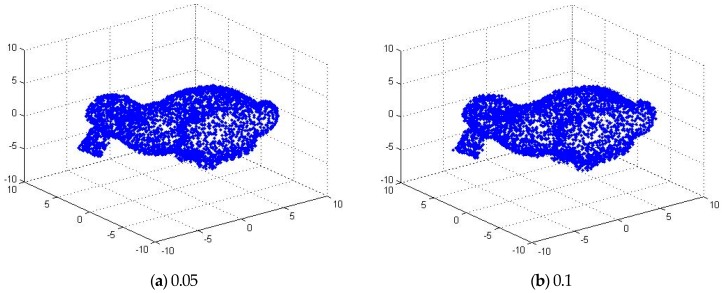
The point cloud data after adding the noise.

**Figure 13:**
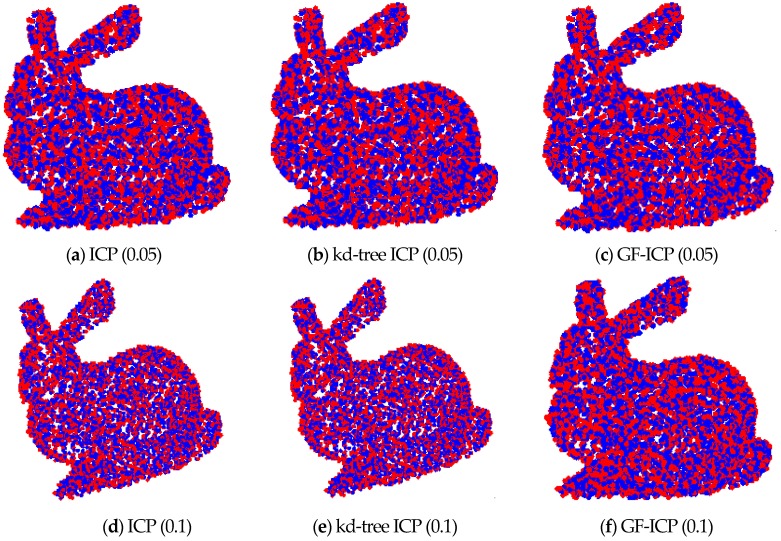
The point cloud registration results of the three algorithms after adding noise.

In order to analyze the immunity of the algorithm to noise, we first added different degrees of noise to the point cloud data. Then, the ICP algorithm based on quaternion, the ICP algorithm based on k-d-tree and the algorithm proposed in this paper are used to match the above point cloud data with different degrees of noise. Figure 12 shows two “bunny” point cloud data superimposed by various levels of zero mean additive Gaussian noise. Among them, the first added variance is 0.05 m, and the second one is 0.1 m. Figure 13 shows the matching results of the three ICP algorithms after applying the Figure 5a bunny point cloud position. Among them, the first row shows the point cloud registration results after adding Gaussian noise with the variance of 0.05 m; the second row shows the point cloud registration results after adding Gaussian noise with the variance of 0.1 m. After the registration of the point cloud, when the noise is 0.05 m, the matching error of the ICP algorithm is 28.6418 m and the iteration number is 21, while the matching error of the GF-ICP algorithm is 28.6438 m and the iteration number is 5. When the noise is 0.1 m, the matching error of the ICP algorithm is 89.5296 m and the iteration number is 25, while the matching error of the GF-ICP algorithm is 89.5574 m and the iteration number is 7. 

It can be seen from the results that the algorithm we proposed in this paper can still get satisfactory registration results when the cloud data contains noise. With the same matching error, the number of iterations required is less than other ICP algorithms.

### 5.4. Real Large-Scale Data Analysis

**Figure 14:**
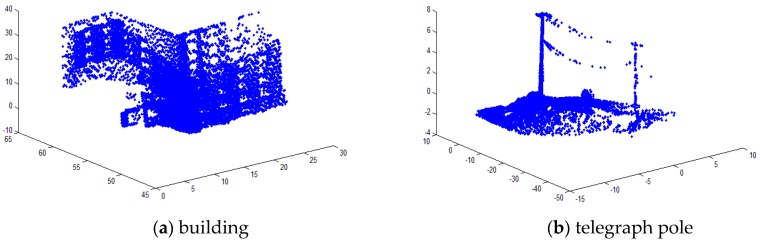

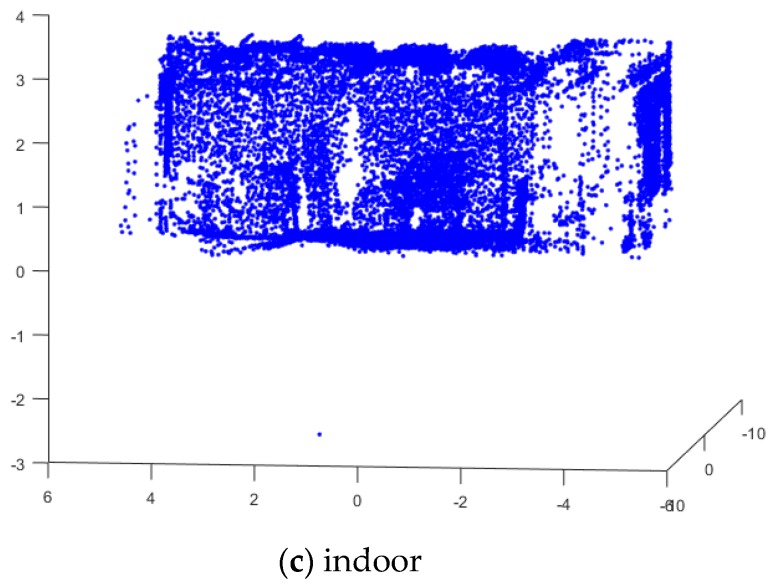
Tested point cloud.

**Figure 15:**
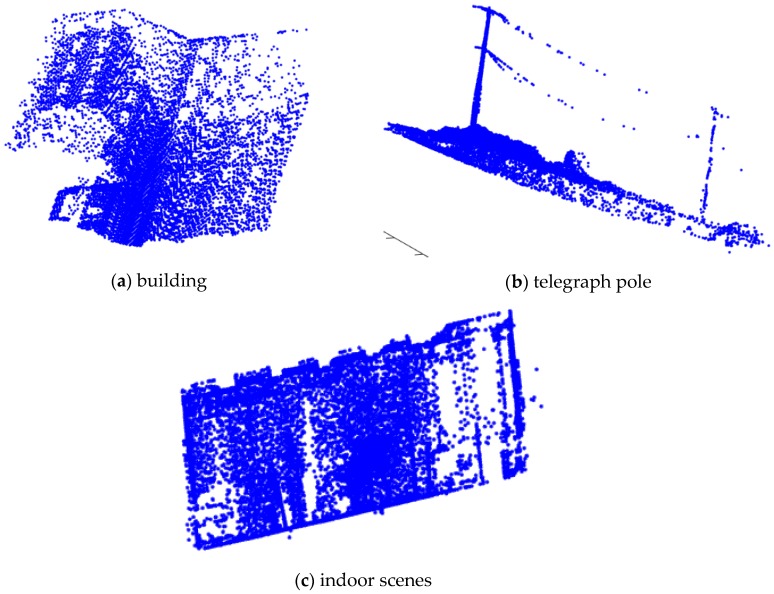
Point cloud registration results.

In order to verify the effectiveness of the algorithm in real large-scale point cloud data registration, we tested different point cloud data. Figure 14 shows three sets of point cloud data to be tested. Figure 14a was a building point cloud dataset [32] provided by the CSDN, the point cloud data is 13292 after down-sampling. Figure 14b was an outdoor telegraph pole point cloud dataset [33] provided by the CSDN, the point cloud data is 17223 after down-sampling. Figure 14c was an indoor scenes point cloud dataset provided by the ASL Datasets [34], the point cloud data is 16496 after down-sampling. These three sets of point cloud data are obtained by scanning real objects with different features. We using MATLAB to randomly generate three sets rotation and translation for the point cloud data. Figure 15 shows the registration results. In Figure 15a, the matching error of the GF-ICP algorithm is 0.7629 m; in Figure 15b, the matching error of the GF-ICP algorithm is 0.9473 m; in Figure 15c, the matching error of the GF-ICP algorithm is 0.9071 m. Figure 15 shows the algorithm proposed in this paper achieve better registration results in large-scale real point cloud data.

## 6. Conclusions 

The ICP algorithm is the mainstream algorithm in the process of accurate registration of 3D point cloud data. However, the algorithm has some problems. It requires a proper initial value and the approximate registration of two point clouds to prevent the algorithm from falling into into a local extremum, but in the actual point cloud matching process, it is difficult to ensure compliance with this requirement. In this paper, we proposed an ICP algorithm based on point cloud features (GF-ICP). The method uses the geometrical features of the point cloud to be registered, such as curvature, surface normal and point cloud density, to search for the correspondence relationships between two point clouds and introduces the geometric features into the error function to prevent the ICP algorithm from falling into a local extremum and achieve accurate registration of two point clouds. Through experimental comparisons, we show that the algorithm proposed in this paper requires less iteration time and has a larger convergence range with the same registration error, which is suitable for the occasions where the initial positions of point cloud are relatively different. In the case of less noise, this algorithm can also accurately realize point cloud registration.

## Figures and Tables

**Table 1:** Run time for the three ICP algorithms.

Method	Run Time (s)		
	Set 1	Set 2	Set 3
ICP	260.347	145.513	263.863
kd-tree ICP	9.456	11.342	8.793
GF-ICP	137.832	135.305	134.320

**Table 2:** Point cloud registration error and the number of iterations required.

Data	ICP		GF-ICP	
	Registration Error (m)	Number of Iterations	Registration Error (m)	Number of Iterations
1	8.0336 × 10^−6^	14	1.665 × 10^−6^	1
2	8.2392 × 10^−6^	21	1.692 × 10^−6^	1
3	8.3355 × 10^−6^	28	1.713 × 10^−6^	1
4	8.7088 × 10^−6^	33	1.759 × 10^−6^	1
5	8.9238 × 10^−6^	54	1.786 × 10^−6^	1
6	1.1598 × 10^4^	78	1.834 × 10^−6^	2
7	8.4294 × 10^3^	100	1.837 × 10^−6^	3
8	8.4351 × 10^3^	100	1.844 × 10^−6^	3
9	7.0844 × 10^3^	100	1.816 × 10^−6^	3
